# Population pharmacokinetics of vancomycin in non-extremely preterm neonates based on real-world studies: influence of daily fluid input and diuretics

**DOI:** 10.1128/spectrum.02274-24

**Published:** 2025-05-14

**Authors:** Kai Zhao, Fang Zhao, KangLu Ju, Hui Chen, Xin Zhai, Ying Chang, ZhenGuo Liu

**Affiliations:** 1 Department of Pharmacy, Northwest Women’s and Children’s Hospital 117943 , Xi'an, Shaanxi, China; University of Iowa, Iowa City, Iowa, USA

**Keywords:** neonates, vancomycin, pharmacokinetic model, therapeutic drug monitoring, model-informed precision dosing

## Abstract

This study aimed to develop a population pharmacokinetics (PPK) model for vancomycin in non-extremely preterm neonates hospitalized in the neonatal intensive care unit (NICU), identify key factors affecting vancomycin pharmacokinetics in this patient population, and formulate an initial dosing protocol. A cohort of 126 neonates admitted to the NICU and treated with vancomycin at Northwest Women’s and Children’s Hospital from January 2019 to December 2023 were included in the study, resulting in the collection of 276 vancomycin concentration values. A PPK model for vancomycin was constructed using a nonlinear mixed-effects approach. The predictive power and stability of the final model were assessed through visual predictive checks, normalized prediction distribution errors, and bootstrapping. Serum creatinine (Scr) level, body weight, daily fluid input, and diuretic usage were identified as significant covariates affecting vancomycin clearance. Utilizing Monte Carlo simulations with the established model, initial recommended dosing regimens for neonates with varying Scr levels, daily fluid input, and diuretic use were estimated. The pharmacokinetic-pharmacodynamic model developed for vancomycin in non-extremely preterm neonates in the neonatal intensive care unit in this study may provide a theoretical reference for research on individualized medication.

**IMPORTANCE:**

A population pharmacokinetic model for vancomycin in neonatal intensive care unit neonates was developed. Daily fluid input and the use of diuretic agents were identified as new significant covariates influencing drug clearance. Based on these covariates, a dosing regimen was developed that provides clinicians with individualized dosing recommendations.

## INTRODUCTION

Methicillin-resistant *Staphylococcus aureus* (MRSA), a clinically prevalent drug-resistant bacterium, has experienced a substantial increase in infection cases globally since its initial identification. Indeed, it has emerged as one of the major pathogens responsible for nosocomial infections, presenting a significant threat to pediatric patients, especially neonates (1). Recent surveillance data on bacterial resistance among pediatric populations in China revealed a persistently high detection rate of MRSA (2, 3). Notably, the incidence of MRSA infections is substantially escalating in neonatal intensive care units (NICUs), where it predominantly leads to conditions such as neonatal sepsis, neonatal purulent meningitis, and neonatal pneumonia.

Vancomycin, a glycopeptide antibiotic, remains one of the most extensively utilized antibiotics for the treatment of severe gram-positive coccal infections. It has a narrow therapeutic index and considerable inter-individual variability in pharmacokinetics. Elevated plasma concentrations of vancomycin are strongly associated with ototoxicity and nephrotoxicity, whereas subtherapeutic concentrations may result in limited efficacy or the development of bacterial resistance. Considering the challenges related to the safety and efficacy of vancomycin therapy in neonates, there is a pressing need to optimize vancomycin exposure in this patient cohort.

Existing evidence suggests that body weight, postmenstrual age, and serum creatinine levels are significant covariates influencing vancomycin clearance (CL) and volume of distribution (V) in population pharmacokinetic (PPK) models for neonates (4, 5). Investigations focusing on various subgroups have further concluded that fluid infusion volume and albumin levels may also serve as important covariates (6, 7). A PPK study conducted on neonates in Chinese neonatal intensive care units identified body weight and serum creatinine as critical covariates (8). However, a limitation of this study was the limited sample size, which precluded the identification of specific covariates unique to the NICU population. In contrast, a study focusing on French NICU neonates identified serum cystatin C levels and mechanical ventilation as potential covariates influencing vancomycin clearance (9). Furthermore, studies conducted on adult populations have indicated that low serum albumin levels and the concomitant use of dopamine may also impact vancomycin clearance in ICU patients (10, 11). Therefore, the purpose of this study was to further elucidate the pharmacokinetic characteristics of vancomycin in non-extremely preterm neonates in the NICU, establish an appropriate PPK model, and develop individualized dosing regimens based on this model.

## MATERIALS AND METHODS

### Study design

Data were collected from pediatric patients admitted to the NICU and treated with vancomycin between January 2019 and December 2023. The inclusion criteria were as follows: (i) postnatal age ≤28 days; (ii) at least one vancomycin concentration value available; and (iii) received vancomycin treatment for ≥3 days. The exclusion criteria were (i) vancomycin concentrations exceeding the detection range (<2 or >50 mg/L); (ii) serum creatinine levels within 7 days after the birth; (iii) patients with congenital renal dysplasia; and (iv) patients with renal dysfunction (chronic or acute renal insufficiency with or without renal replacement therapy).

The fundamental data obtained from the patients encompassed (i) demographic characteristics, including gender, mode of delivery (vaginal/cesarean section), gestational age (GA) at delivery, Apgar score (1 minute, 5 minutes, and 10 minutes), postnatal age (PNA) at admission, birthweight (BW), weight (WT) at the start of vancomycin medication, postmenstrual age (PMA) at the start of vancomycin medication, diagnosis at admission, site of infection at the time of vancomycin treatment, and the neonatal critical illness score (NCIS) at the time of admission (12). The scoring system primarily selects the most abnormal values within 24 hours for heart rate, blood pressure, respiratory rate, PaO_2_, pH, Na^+^, K^+^, Cr/BUN, hematocrit, and gastrointestinal manifestations, comprising a total of 10 parameters. Each parameter is scored out of 10 points. A total score exceeding 90 indicates non-critical status, a score between 70 and 90 signifies critical status, and a score below 70 denotes extremely critical status. (ii) Laboratory parameters, such as the levels of serum creatinine (Scr), albumin (ALB), alanine aminotransferase (ALT), aspartate aminotransferase (AST), and blood urea nitrogen (BUN). (iii) Vancomycin-related information, including the date of vancomycin administration, administration time, sampling time, plasma concentration levels, and daily dosage. (iv) Concomitant medications and treatments at the start of vancomycin medication, including diuretics (e.g., furosemide, spironolactone, and hydrochlorothiazide), vasoactive drugs (e.g., dopamine, dobutamine, epinephrine, and norepinephrine), non-steroidal anti-inflammatory drugs (e.g., ibuprofen and indomethacin), human serum albumin, piperacillin-tazobactam, respiratory support (including oxygen therapy and mechanical ventilation), daily fluid input (DFI), daily urine volume (DUV), and mild hypothermia therapy.

Vancomycin, sourced from Suzhou Lilly Pharmaceutical Factory, was administered at a dosage of 10–15 mg/kg of body weight per dose, with dosing intervals ranging from every 12 hours to every 8 hours. The standard infusion duration for the clinical administration of vancomycin is 1 hour, with trough and peak concentrations assessed 0.5 hours prior to and following the infusion, respectively. All measured concentrations reflect steady-state levels achieved after four doses. Blood samples were anticoagulated using ethylenediaminetetraacetic acid, centrifuged at 1,000 rpm for 5 minutes, and subsequently analyzed via chemiluminescence immunoassay (VIVA, Siemens). Calibration and quality control of samples were performed in accordance with the manufacturer’s instructions, with a calibration range of 2–50 mg/L.

### Establishment of the PPK model

In the current study, a PPK model was developed utilizing the nonlinear mixed effects modeling approach. The first-order conditional estimation method with extended least squares was employed to estimate the pharmacokinetic parameters and their variability. The modeling process was conducted using Phoenix NLME software (version 8.3.5.340; Certara, St. Louis, MO, USA).

#### Base model

The pharmacokinetic characteristics of vancomycin were characterized using both one-compartment and two-compartment models with first-order elimination. The exponential variability error model was utilized to evaluate the inter-individual variability of structural model parameters (equation 1). For each model, additive, proportional, and combined additive/proportional error models were examined to address residual variability (equations 2-4).


(1) $$P_{i}=P_{TV}\times e^{\eta i},$$

(2) $$Y=F+\varepsilon_{1},$$

(3) $$Y=F\times (1+\varepsilon_{1}),$$

(4) $$Y=F\times (1+\varepsilon_{1})+\varepsilon_{2}.$$

In these equations, *P*_TV_ denotes the population typical value of pharmacokinetic parameters, *P*_*i*_ represents the pharmacokinetic parameter for the *i*th individual, and *ηi* signifies the random effect for the *i*th individual. *Y* stands for the observed value, *F* denotes the individual predicted value, and *ε* signifies the residual variability, which is symmetrically distributed with a mean of 0 and a variance of *σ*².

#### Covariate models

Continuous covariates encompassed body weight, PMA, Scr, ALB, ALT, AST, BUN, DFI, and DUV. Categorical covariates included gender (female/male), admission NCIS status (>90/70–90/< 70), respiratory support (yes/no), mild hypothermia therapy (yes/no), and concomitant medications (yes/no). These covariates were evaluated to determine their influence on vancomycin clearance and volume of distribution.

Based on prior research findings from the vancomycin maturation model in NICU neonates (8), an exponential model was used to evaluate the influence of covariates on pharmacokinetic parameters using a stepwise forward addition and backward elimination methodology. During the forward addition phase, a covariate was deemed to significantly affect the model parameters if the reduction in the objective function value (OFV) exceeded 3.84 (*P* < 0.05, df = 1). In the backward elimination phase, a covariate was deemed to exert a significant influence on model parameters and was consequently retained if the increase in the OFV exceeded 7.88 (*P* < 0.005, df = 1). Conversely, if this criterion was not met, the covariate was excluded from the model.

#### Model evaluation

The diagnostic goodness-of-fit plots were subjected to visual inspection to validate the selection of the final model. Model fit was evaluated by examining the concordance between observed values (DV) and individual predicted values (IPRED), as well as between DV and population predicted values (PRED). Additionally, the uniformity of the distribution of conditional weighted residuals (CWRES) in relation to PRED and time after dose was assessed. To further examine the stability and predictive performance of the final model, the bootstrap method and normalized prediction distribution errors (NPDE) were employed. Utilizing the bootstrap method, a total of 5,000 data sets were generated via repeated sampling from the original data set. Subsequently, the model parameters for each of these data sets were computed and documented. The median and the 95% confidence interval (ranging from 2.5% to 97.5%) of these estimated parameters were then calculated and compared against the final model estimates. Monte Carlo simulations were implemented using the R software package (version 2.0, http://www.npde.biostat.fr/) to generate (i) quantile-quantile plots of NPDE, (ii) histograms of NPDE, (iii) scatter plots of NPDE versus time, and (iv) scatter plots of NPDE versus PRED were generated to summarize the NPDE results. If the predictive performance is satisfactory, NPDE follows a normal distribution (Shapiro-Wilk test), with a mean of 0 (*t*-test) and a variance of 1 (Fisher test).

#### Simulation and dosage recommendation

The finally established population pharmacokinetic model was used to establish a vancomycin dosing regimen for achieving the pharmacodynamic target of AUC24h/MIC ≥ 400 in neonates. For MIC = 1 mg/L, the daily dose can be calculated using the final model with estimated clearance and equation 5.


(5) Dose=400×CL.

Virtual patients were simulated with varying covariate parameters to identify the most appropriate regimen to meet the target. Each regimen underwent 5,000 simulations using the Monte Carlo method, executed via the Crystal Ball (11.1.2.2) software in Microsoft Excel.

## RESULTS

### Characteristics

A total of 126 patients and 276 vancomycin concentration data were included. Among them, the data of 112 patients (from January 2019 to June 2023) were used for modeling, while those of 14 patients (from July 2023 to December 2023) were used for validation. Of the included patients, 68.3% were male, and only 38.1% were delivered vaginally. The median gestational age and PMA were 32 and 35.7 weeks, respectively, while the median birth weight and weight at the time of vancomycin administration were 1.45 and 1.98 kg, respectively. Additionally, 73.8% (93/126) of the patients were preterm infants, and the majority (95/126) had bloodstream infections. The characteristics of the patients and the comparative analysis between groups are presented in Table 1. Given that only a small number of patients received mild hypothermia therapy (5/126), nonsteroidal anti-inflammatory drugs (3/126), or cimetidine (3/126), these factors were not considered covariates. A significant difference between the two groups was observed only in the NCIS score (*P* = 0.038).

**TABLE 1:** Summary of the baseline information^*l*^

Item	All patients (*N* = 126)	Modeling group (*N* = 112)	Validation group (*N* = 14)	*P* value
	*N* (%) or median (IQR^*a*^)	*N* (%) or median (IQR^*a*^)	*N* (%) or median (IQR^*a*^)	
Gender (male)	86 (68.3)	78 (69.6)	8 (57.1)	0.370
Mode of delivery (vaginal)	48 (38.1)	44 (39.3)	4 (28.6)	0.436
GA at delivery (weeks)	32 (29.38–37.85)	32 (29.08–37.63)	32.4 (30.3–39.05)	0.664
1-minute Apgar score	8 (7–9)	8 (7–9)	8.5 (7.3–9)	0.303
5-minute Apgar score	9 (8–10)	9 (8–10)	9 (8.3–10)	0.395
10-minute Apgar score	9 (8–10)	9 (8–10)	9.5 (9–10)	0.151
PNA at admission (days)	0^*j*^(0–2.8)	0 (0–2)	1 (0–4)	0.159
PMA (weeks)	35.7 (32.9–39.98)	35.6 (32.98–39.9)	36.65 (32.68–41.45)	0.624
BW (kg)	1.45 (1.14–2.88)	1.45 (1.14–2.65)	1.51 (1.14–3.35)	0.617
WT (kg)	1.98 (1.35–2.98)	1.96 (1.35–2.94)	2.03 (1.31–3.63)	0.377
Diagnosis at admission				
Prematurity^*b*^	93 (73.8)	83 (74.1)	10 (71.4)	0.759
ELBW	23 (18.3)	22 (19.6)	1 (7.1)	0.463
VLBW	44 (34.9)	38 (33.9)	6 (42.9)	0.558
LBW	20 (15.9)	19 (17.0)	1 (7.1)	0.697
Respiratory^*c*^	78 (61.9)	69 (61.6)	9 (64.3)	0.846
Gastrointestinal^*d*^	10 (7.9)	9 (8)	1 (7.1)	0.680
Twin or multiple pregnancy	22 (17.5)	19 (17)	3 (21.4)	0.734
Cardiovascular^*e*^	2 (1.6)	2 (1.8)	0 (0)	1
Neurological^*f*^	12 (9.5)	9 (8)	3 (21.4)	0.680
Metabolic^*g*^	5 (4)	5 (4.5)	0 (0)	1
Infections^*h*^	30 (23.8)	25 (22.3)	5 (35.7)	0.153
Infection site				0.555
Bloodstream	95 (75.4)	86 (76.8)	9 (64.3)	
Neural	32 (25.4)	25 (22.3)	7 (50)	
Other^*i*^	21 (16.7)	20 (17.9)	1 (7.1)	
Vancomycin concentrations (trough)	143 (51.8)	129 (51.2)	14 (58.3)	0.503
Diuretics	42 (33.3)	40 (35.7)	2 (14.3)	0.139
Vasoactive drugs	40 (31.7)	36 (32.1)	4 (28.6)	0.795
Human albumin	25 (19.8)	22 (19.6)	3 (21.4)	0.777
Piperacillin-tazobactam	12 (9.5)	12 (10.7)	0 (0)	0.358
Respiratory support	38 (30.2)	33 (29.5)	5 (35.7)	0.758
NCIS Score				0.038^*k*^
>90	10 (7.9)	10 (8.9)	0 (0)	
70–90	88 (69.9)	81 (72.3)	7 (50)	
<70	28 (22.2)	21 (18.8)	7 (50)	
Scr (µmol/L)	32.48 (24.54–42.31)	32.31 (24.42–41.76)	32.71 (28.05–43.09)	0.937
ALB (g/L)	28.5 (25.07–31.55)	28.23 (24.76–31.44)	30.47 (28.06–31.69)	0.087
ALT (U/L)	10.69 (6.46–15.53)	10.84 (6.45–16.61)	10.46 (6.56–13.71)	0.589
AST (U/L)	28.95 (21.38–41.9)	29.64 (21.25–41.9)	26.13 (21.72–39.67)	0.502
BUN (mmol/L)	3.57 (2.31–5.86)	3.62 (2.39–6.03)	3.28 (1.91–4.34)	0.272
Daily fluid input (mL)	364 (252.91–473.1)	365.15 (255.64–478.1)	364 (243–397.88)	0.828
Daily urine volume (mL)	200.5 (156.75–283.25)	213.5 (160–290)	230 (141.43–345.75)	0.704
Initial dose (mg)	25 (18–36.75)	25 (18–35)	25.5 (14.5–41.6)	0.994
Vancomycin concentration (mg/L)	16.5 (10–24.3)	17.1 (10–24)	14.7 (10.2–25.75)	0.912
Mild hypothermic therapy	5 (4)	5 (4.5)	0 (0)	0.448
Cimetidine (yes/no)	3 (2.4)	3 (2.7)	0 (0)	0.549
NSAIDs (yes/no)	3 (2.4)	3 (2.7)	0 (0)	0.549

^*a*^ IQR is the interquartile range.

^*b*^ Premature infants are defined as infants born alive before 37 weeks gestation.

^*c*^ Including transient apnea of the newborn, transient tachypnea of the newborn, meconium aspiration syndrome, respiratory distress syndrome, asphyxia, pulmonary hemorrhage, and respiratory failure.

^*d*^ Including necrotizing enterocolitis, intestinal perforation, gastrointestinal bleeding, Hirschsprung's disease, and neonatal intestinal obstruction.

^*e*^ Persistent pulmonary hypertension of the newborn.

^*f*^ Including intracranial hemorrhage, neonatal hypoxic-ischemic encephalopathy, hydrocephalus, and tethered spinal cord syndrome.

^*g*^ Including neonatal jaundice, metabolic acidosis, and hyperlactatemia.

^*h*^ Including sepsis, meningitis, necrotizing enterocolitis, and pneumonia.

^*i*^ Other infection sites include the urinary system, digestive system, and respiratory system.

^*j*^ For postnatal stages, the day of birth was designated postnatal day 0.

^*k*^ *P*＜0.05.

^*l*^ ELBW, extremely low birth weight; VLBW, very low birth weight; LBW, low birth weight; and NSAIDs, non-steroidal anti-inflammatory drugs.

### Model establishment

The examination of residual variability supported the adoption of a proportional error model. Upon the establishment of the base model, the outcomes of the covariate selection are presented in Table 2. Compared to the base model, body weight emerged as the most crucial covariate for vancomycin clearance and volume of distribution. In the final model, body weight, serum creatinine level, daily fluid input, and diuretic use significantly impacted vancomycin clearance, whereas body weight significantly influenced vancomycin volume of distribution. The final model for vancomycin clearance is represented by equation 6, and the final model for volume of distribution is represented by equation 7.

**TABLE 2:** Screening process of covariates^*a*^

Steps	Covariates	∆OFV
Univariate screening		
Covariate influence on CL	SEX	−0.169
	PM	−36.347
	WT	−150.148
	PMA	−113.109
	SCR	−7.215
	BUN	−1.022
	ALB	−18.17
	ALT	−5.607
	AST	−0.003
	DFI	−20.894
	DA	−4.609
	NCIS	−2.626
	RS	−5.495
	PTZ	−0.001
	HA	0
	VAA	−1.992
	DUV	−48.043
Covariate influence on V	SEX	−1.965
	PM	−20.705
	WT	−73.992
	PMA	−39.657
	SCR	−1.708
	BUN	−0.023
	ALB	−8.144
	ALT	−1.482
	AST	−0.943
	DFI	−18.12
	DA	−0.01
	NCIS	−1.831
	RS	−5.828
	PTZ	−0.001
	HA	−5.145
	VAA	−0.348
	DUV	−23.771
Stepwise inclusion for multivariate analysis		
Step 1, WT for CL	WT for V	−115.634
Step 2, WT for CL and V	DA for Cl	−15.724
Step 3, WT and DA for CL, WT for V	HA for V	−6.709
Step 4, WT and DA for CL, WT and HA for V	BUN for V	−6.347
Step 5, WT and DA for CL, WT, BUN, and HA for V	PTZ for Cl	−5.639
Step 6, WT, DA, and PTZ for CL, WT, BUN, and HA for V	PMA for Cl	−4.543
Step 7, WT, DA, PTZ, and PMA for CL, WT, BUN, and HA for V	DFI for Cl	−4.788
Step 8, WT, DA, PTZ, PMA, and DFI for CL, WT, BUN, and HA for V	Scr for Cl	−7.869
Step 9, WT, DA, PTZ, PMA, DFI, and SCR for CL, WT, BUN, and HA for V	No effect chosen to add	
Stepwise backward elimination for multivariate analysis		
Step 1, WT, DA, PTZ, PMA, DFI, and SCR for CL, WT, BUN, and HA for V	PMA for Cl	2.804
Step 2, WT, DA, PTZ, DFI, and SCR for CL, WT, BUN, and HA for V	PTZ for Cl	5.458
Step 3, WT, DA, DFI, and SCR for CL, WT, BUN, and HA for V	BUN for V	6.047
Step 4, WT, DA, DFI, and SCR for CL, WT and HA for V	HA for V	5.651
Step 5, WT, DA, DFI, and SCR for CL, WT for V	No effect chosen to subtract	

^*a*^ PM, preterm infant; RS, respiratory support; PTZ, piperacillin-tazobactam; HA, human albumin; and VAA, vasoactive agents.


(6) $$CL(L/h)=0.14\times (WT/2.12)\times (Scr/30.52)\times (DFI/367.18)\times e^{A}\times exp^{\left(\eta CL\right)},$$

(7) V=1.04×(WT/2.12).

When diuretics were used concomitantly, A = −0.20, and when diuretics were not used, A = 0.

**Fig 1:**
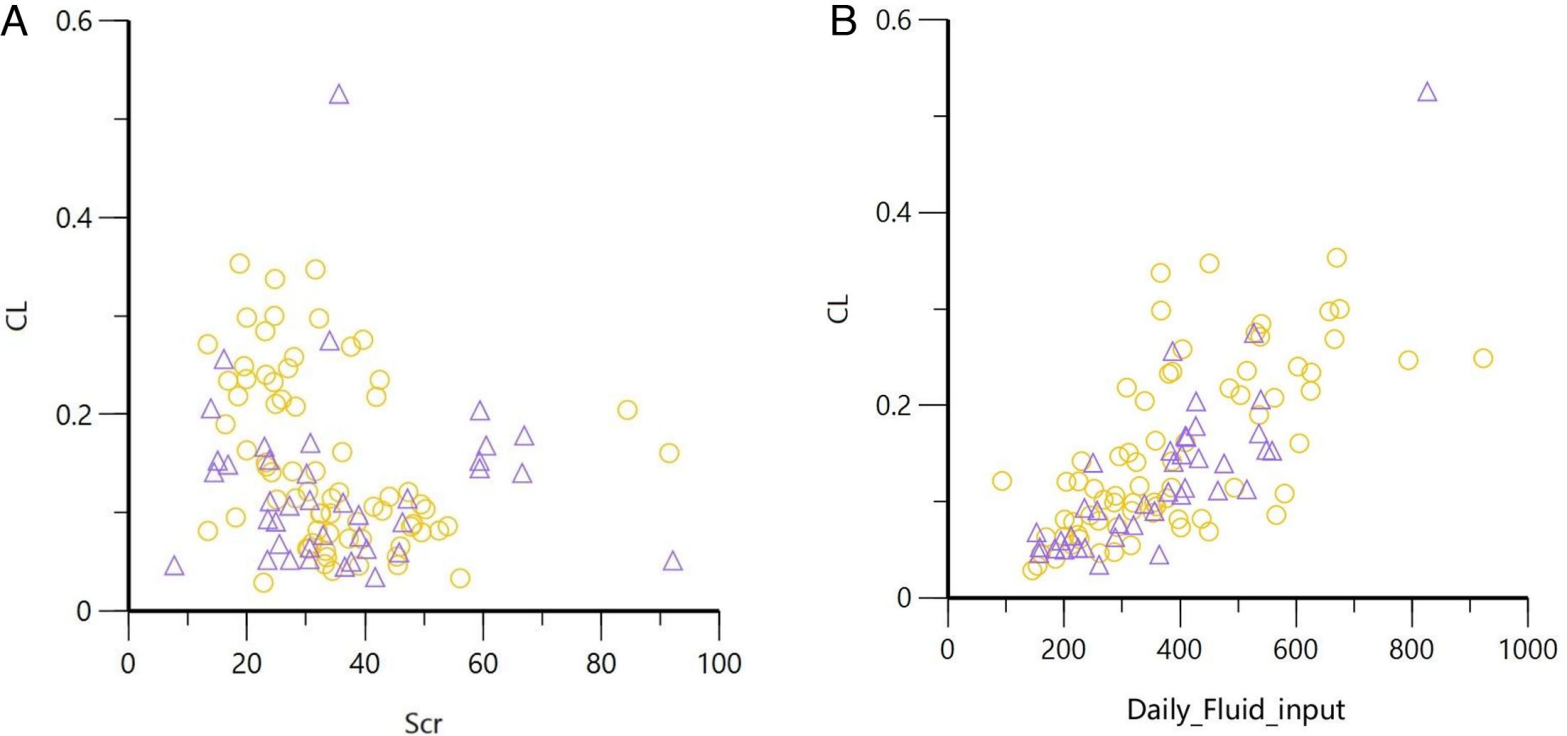
Scatter plots illustrating the relationships between Scr, DFI, and CL in patients with and without DA Use. “〇” denotes patients not using diuretics, while “△” denotes patients using diuretics. (A) The relationship between Scr and CL. (B) The relationship between DFI and CL.

Moreover, the relationship between Scr and DFI with vancomycin CL was assessed in patients who did and did not use diuretics (Fig. 1).

The final model indicated a typical CL value of 0.14 L/hour. Compared to the base model, the random inter-individual variability in CL in the final model was significantly lower (24.84% versus 4.97%). Table 3 summarizes the parameter estimates, relative standard errors, 95% confidence intervals, inter-individual variability, and intra-individual residual variability results for the base model, final models, and bootstrap.

**TABLE 3:** Pharmacokinetic parameter estimates for the final model and bootstrap results^*a*^

Parameters	Base model (RSE%)	Final model (RSE%)	Bootstrap (95% CI)
Population parameters			
CL (L/hour)	0.12 (5.98)	0.14 (3.15)	0.14 (0.13–0.15)
WT on CL (L/hour)		1.13 (5.64)	1.15 (1.01–1.36)
Scr on CL (L/hour)		−0.15(−31.34)	−0.15 (−0.25 to −0.017)
DFI on CL (L/hour)		0.14 (27.03)	0.13 (−0.087 to −0.21)
DA on CL (L/hour)		−0.20 (−20.03)	−0.20 (−0.39 to −0.051)
V (L)	0.95 (6.57)	1.04 (4.28)	1.03 (0.97–1.10)
WT on V (L)		1.07 (7.61)	1.07 (0.92–1.22)
Inter-individual variability parameter			
CL (%CV)	24.84 (15.70)	4.97 (15.70)	4.80 (2.83–6.77)
V (%CV)	0.90 (215.55)		
Residual variability parameter			
Proportional (%CV)	28.3 (6.85)	18.0 (5.76)	17.6 (14.81–20.69)

^*a*^ RSE%, relative standard error percentage; 95% CI, 95% confidence interval; V, apparent volume of distribution of vancomycin; and %CV, coefficient of variation.

### Model evaluation

#### Diagnostic plots

**Fig 2:**
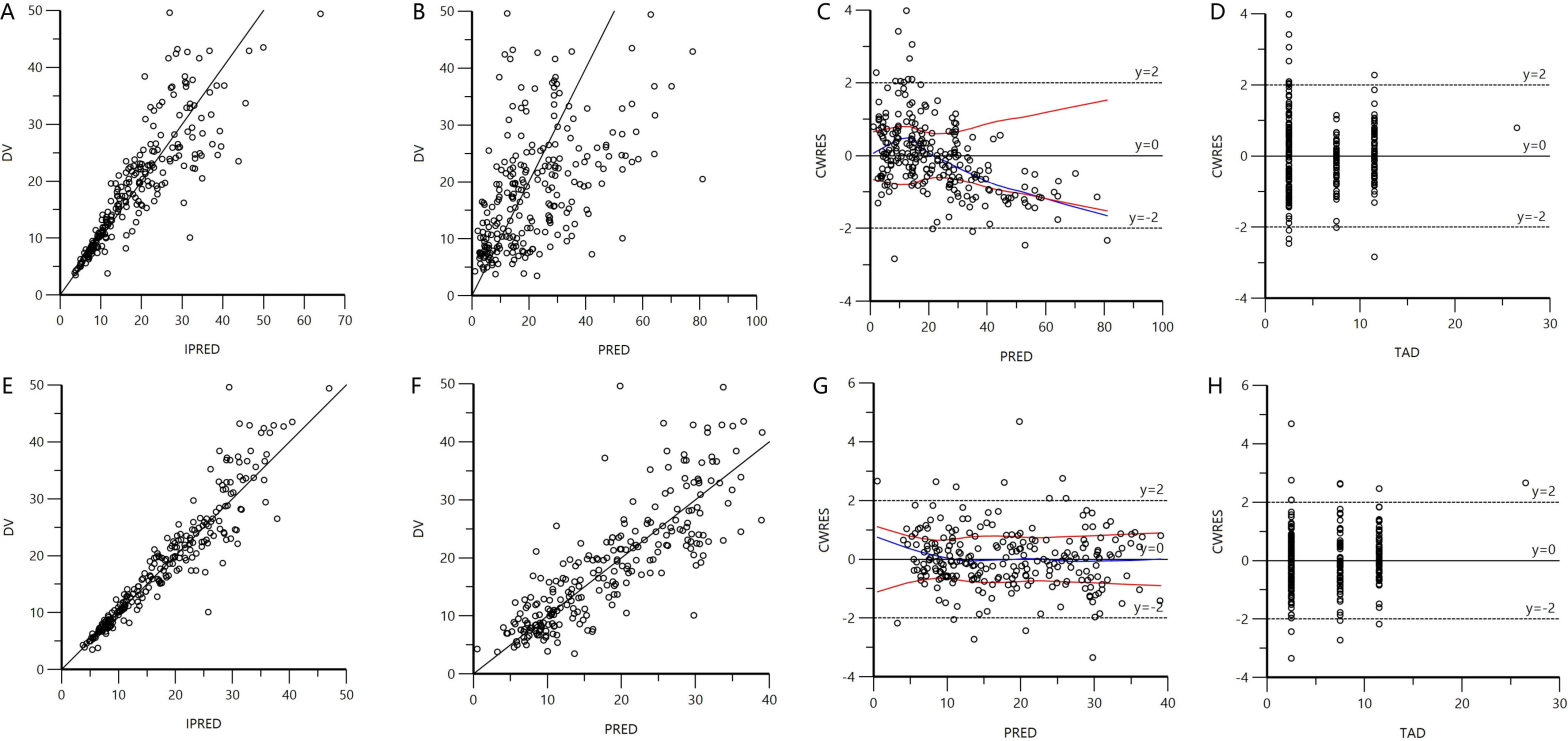
Diagnostic fit plots for the base model (A through D) and final model (E through H). (A and E) Comparison of IPRED versus observed concentrations. (B and F) Population predicted concentrations versus observed concentrations. (C and G) PRED versus conditionally weighted residuals. (D and H) Time after dose versus CWRES.

Compared to the base model, data fitting was significantly superior in the final model, as evidenced by the absence of deviations or substantial trends in the scatter plots. The goodness-of-fit plots for both the base and final models are illustrated in Fig. 2.

#### Bootstrap

The final model underwent validation utilizing a 1,000-iteration bootstrap method, with the results detailed in Table 3. As anticipated, the medians and distributions of parameters derived from the bootstrap procedure were closely aligned with the estimated values from the final PPK model. Furthermore, all parameter values of the final model fell within the 95% confidence intervals of the corresponding bootstrap-derived parameter values, highlighting the stability of the final model and the accuracy of its parameter estimates.

#### Visual predictive check

**Fig 3:**
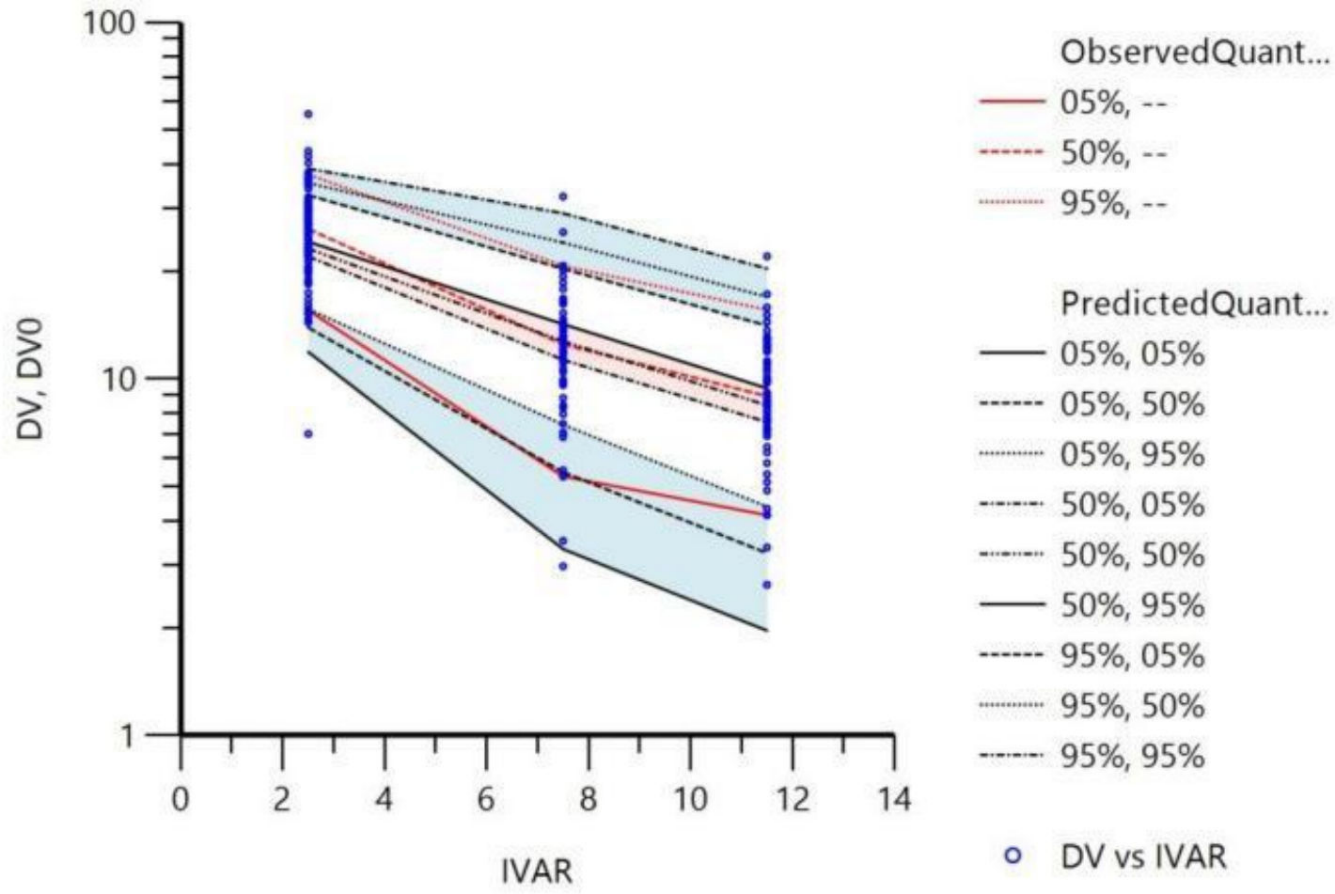
Results of the VPC of the final model. DV, dependent variable (drug concentration); and IVAR, independent variable (time after dosing).

A visual predictive check (VPC) was performed on the final model utilizing 1,000 simulations, with the results depicted in Fig. 3. The analysis involved comparing the 5th, 50th, and 95th percentiles of the observed data with those of the simulated data, alongside the 95% confidence intervals for the corresponding percentiles of the simulated data. The findings revealed that the three quantile curves (in red) of the observed data were well within the respective prediction intervals of the simulated data, indicating a satisfactory fit of the simulation model.

#### Normalized prediction distribution error

**Fig 4:**
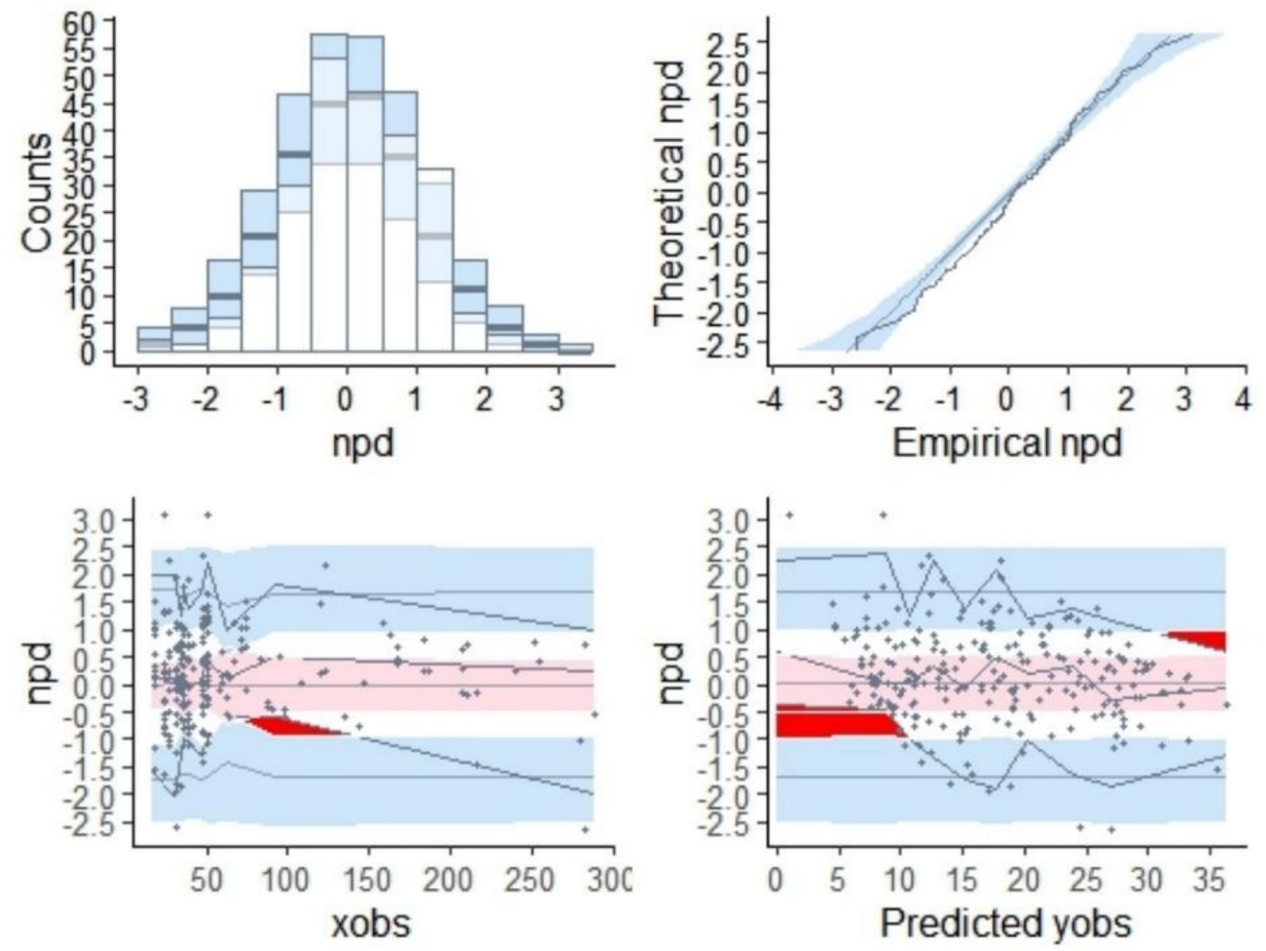
NPDE plots.

The distribution of normalized prediction distribution errors for the final model is presented in Fig. 4. The *t*-test resulted in a *P*-value of 0.0723, while Fisher’s variance test yielded a *P*-value of 0.0711. Additionally, the Shapiro-Wilk test for normality returned a *P*-value of 1. The overall adjusted *P*-value was 0.0711, indicating homogeneity of variance and robust predictive performance of the model, thereby validating its suitability for generating simulated data.

#### External validation

When the 24 blood concentration values from the 14 pediatric patients utilized for model validation were incorporated into the final model for prediction error testing, the resulting metrics for mean prediction error, mean absolute prediction error, fraction within 20%, and fraction within 30% (F30%) were 2.74%, 17.48%, 75.00%, and 83.33%, respectively. These external validation results collectively demonstrated the robust predictive performance of the final model.

### Model application

Based on the final model, a patient weight range of 1–5 kg was simulated, following which Monte Carlo simulations were used to develop a recommended initial dosing table for vancomycin in non-extremely preterm neonates within the NICU. This dosing table took into account varying levels of Scr, DFI, and the administration of DA. The resulting dosing recommendations are detailed in Table 4.

**TABLE 4:** Recommended initial dose of vancomycin in the NICU

Scr (µmol/L)	DFI (mL)	Dose (mg/kg/day)	
		DA	Non-DA
10	100	26	32
	250	29	36
	400	31	38
	550	33	39
	700	34	41
30	100	22	27
	250	25	30
	400	27	32
	550	28	34
	700	29	35
50	100	21	25
	250	23	28
	400	25	30
	550	26	31
	700	27	32
70	100	20	24
	250	22	27
	400	24	29
	550	25	30
	700	26	31
90	100	19	23
	250	21	26
	400	23	28
	550	24	29
	700	25	30

## DISCUSSION

As is well documented, vancomycin is the primary therapeutic agent for the treatment of infections caused by methicillin-resistant gram-positive cocci in neonates within the NICU (13). However, there remains a lack of consensus regarding the optimal dosage and efficacy evaluation criteria for vancomycin administration, as evidenced by existing guidelines (14–16). Furthermore, the diverse pathophysiological conditions of neonates admitted to the NICU contribute to significant variability in the pharmacokinetic parameters of vancomycin among individuals (17). Consequently, determining the appropriate vancomycin dosage for NICU neonates continues to pose a substantial clinical challenge. Herein, a pharmacokinetic model for vancomycin in the neonatal intensive care unit population was developed to identify the optimal initial dosing regimen for this cohort. Our analysis unveiled that weight, serum creatinine, daily fluid input, and diuretic agent significantly influenced vancomycin clearance.

Numerous medication dosage guidelines in both China and other countries (18–21) are based on the child’s age, incorporating parameters such as gestational age, postnatal age, and postmenstrual age. Additionally, some dosing guidelines (22, 23) integrate neonatal age with serum creatinine levels to inform dosing recommendations. However, the recommended dosages frequently lead to suboptimal blood concentrations of vancomycin, with the incidence of subtherapeutic levels ranging from 47% to 93% (24). Furthermore, empirical dosing regimens achieve target vancomycin concentrations in merely 20%–50% of the neonatal population (25).

Several recent studies have investigated the optimal initial dosing of vancomycin in neonates within the NICUs; nonetheless, there remains a lack of universal consensus regarding the identification of key covariates. Li et al. (8) reported that weight and serum creatinine levels significantly influence vancomycin clearance, with no observed racial differences between Asian and Caucasian neonates. In contrast, Lee et al. (5) and Alsultan et al. (26) identified weight, serum creatinine, and postmenstrual age as influential factors affecting neonatal vancomycin clearance. The aforementioned studies indicate that pharmacokinetic models tailored for distinct neonatal populations may not be universally applicable across different groups. Thus, there is an urgent need to develop a population pharmacokinetic model specific to our center and establish an initial dosing regimen.

The clearance of vancomycin, a hydrophilic drug predominantly excreted through renal pathways, is influenced by various factors affecting renal function. Importantly, our findings indicated that the effects of WT and Scr on clearance were consistent with those of earlier studies. Additionally, DFI and DA were identified as significant covariates influencing vancomycin clearance. Specifically, increased daily fluid input may necessitate a higher daily dose, whereas the concomitant use of diuretics may require a lower daily dose. According to a previous study (6), fluid volume in very low birth weight neonates is a significant covariate influencing vancomycin clearance. An increase in fluid volume results in elevated urine output and enhanced glomerular filtration, subsequently increasing the clearance of the hydrophilic drug vancomycin (27). Herein, DFI was used instead of infusion volume as a more accurate measure of fluid balance in NICU neonates. Although daily fluid intake is clinically calculated based on body weight, our correlation analysis revealed that the weight-DFI correlation (*R* = 0.7274) was weaker than the weight-PMA correlation (*R* = 0.8696). This result suggests that, despite a strong correlation between body weight and DFI, DFI still holds clinical significance independent of body weight. The outcomes of our covariate screening were in line with the findings of the aforementioned study. In a previous study (28), DUV was utilized as an indicator for evaluating renal function and pioneering an initial vancomycin dosing regimen for preterm neonates. However, this regimen proved unsuitable for all neonates. In the current study, DUV was also considered a potential influencing factor during covariate screening. On the other hand, our findings indicated that urine volume was not a significant covariate affecting clearance, in agreement with the results of the aforementioned reports.

Diuretics are frequently administered to neonates for the management of edematous disorders, respiratory conditions, and respiratory distress syndrome (29). However, their use is associated with nephrotoxicity. Research indicates (30) that diuretics can bind to antigens in the kidneys or function as antigens deposited in the renal interstitium, thereby inducing acute interstitial nephritis. Furthermore, diuretics may exacerbate renal dysfunction by elevating urine output, which can result in elevated vancomycin exposure and subsequent nephrotoxicity (31). A pharmacokinetic study (32) investigating vancomycin in adult trauma patients recommended that the dosage of vancomycin be reduced when co-administered with furosemide. Additionally, a systematic meta-analysis (33) of risk factors for vancomycin-associated acute kidney injury (AKI) demonstrated that the concurrent use of diuretics elevates the incidence of vancomycin-associated AKI. In this study, the clearance of vancomycin was lower when administered in conjunction with the diuretic furosemide, warranting a reduction in the initial dose of vancomycin. This finding aligns with the results of the aforementioned study.

Furthermore, in addition to WT, Scr, and PMA, other studies have identified additional covariates that significantly impact vancomycin clearance. For instance, Tang et al. (34) reported that the concomitant use of vasoactive drugs may decrease vancomycin clearance, necessitating a reduction in the daily dose and potentially increasing the incidence of acute kidney injury. In the current study, approximately 31.7% (40 out of 126) of the children were concomitantly treated with vasoactive drugs, a proportion marginally higher than the previously reported utilization rate of 21.4% (39 out of 182). However, this factor did not exhibit a significant effect during the covariate screening process. Smits et al. (7) identified albumin level as the most critical covariate for free vancomycin concentration, noting a strong linear correlation between free and total vancomycin concentrations. Free vancomycin concentrations decreased with increasing albumin levels. Besides, vancomycin clearance increased with rising albumin levels during the initial covariate screening herein. However, albumin was not identified as a significant covariate for vancomycin clearance in the subsequent analysis.

Despite developing a pharmacokinetic model for vancomycin tailored to the neonatal population in our center’s NICU, this study has several limitations that cannot be overlooked. To begin, given the single-center retrospective nature of this study, our data were restricted to trough or peak concentrations for each neonate, thereby compromising the generalizability of our model to other centers and warranting rigorous validation before making any clinical recommendations. Second, this study neither identified cases of AKI nor evaluated the relationship between medication administration and clinical outcomes in pediatric patients. Consequently, elucidating the association between vancomycin dosing and both clinical efficacy and nephrotoxicity represents a critical area for future research. Finally, the sample size and covariates included in this study were limited, and the study population did not incorporate extremely preterm infants. Additionally, the NCIS score was assessed at admission rather than at the time of study inclusion, which may have led to the omission of certain significant covariates. Therefore, future research should aim to expand the sample size and adopt a multicenter approach to validate our findings.

### Conclusion

In this study, a population pharmacokinetic model for vancomycin in non-extremely preterm neonates in the NICU was developed. Weight, serum creatinine level, daily fluid input, and use of diuretic agent were identified as significant covariates influencing drug clearance. Based on these covariates, a dosing regimen aimed at achieving an area under the concentration-time curve to a minimum inhibitory concentration ratio of ≥400 was developed, assuming a MIC of 1. This regimen provides clinicians with individualized dosing recommendations. Nevertheless, further research is required to validate the safety and efficacy of this dosing strategy.

## Data Availability

The raw modeling data sets are available at https://doi.org/10.6084/m9.figshare.28882814. Other underlying research data and materials can be accessed by contacting the corresponding author.
